# Understanding Eosinophil Heterogeneity: The Known and Unknown

**DOI:** 10.3390/cells15060564

**Published:** 2026-03-21

**Authors:** Alexander Ruzic, Michael Trus, Roma Sehmi, Manali Mukherjee

**Affiliations:** 1 Department of Medicine, McMaster University, Hamilton, ON L8S 4L8, Canada; ruzica@mcmaster.ca (A.R.); sehmir@mcmaster.ca (R.S.); 2 Department of Pathology and Molecular Medicine, McMaster University, Hamilton, ON L8S 4L8, Canada; trus@hhsc.ca; 3 Research Institute of St. Joe’s, St. Joseph’s Healthcare Hamilton, Hamilton, ON L8N 4A6, Canada

**Keywords:** eosinophil, severe asthma, COPD, eosinophil subpopulations, airway microenvironment, immunophenotype, eosinophil transcriptome

## Abstract

Eosinophils are multifunctional granulocytes with central roles in the pathobiology of chronic airway diseases. While systemic eosinophilia (>300 cells/μL) is a well-established biomarker to guide therapeutic decision-making, accumulating evidence indicates that eosinophils are not a uniform population but instead exhibit substantial phenotypic and functional heterogeneity across biological compartments, inflammatory states, and disease contexts. In this review, we synthesize the current understanding of eosinophil heterogeneity in airway diseases and critically evaluate the strengths and limitations of surface marker-based approaches, with emphasis on CD62L/L-selectin-defined subpopulations. Although CD62L-based stratification has provided valuable insight into eosinophil activation and tissue localization, its limited specificity, inconsistent clinical associations, and reliance on murine models restrict its utility as a framework for eosinophil subtyping in humans. We highlight how transcriptomic and proteomic profiling has transformed the field by revealing that peripheral blood eosinophils are largely quiescent, whereas disease-relevant functional specialization is predominantly acquired within inflamed tissues in response to cues from the local microenvironment. These molecular studies support a model in which eosinophil heterogeneity represents a continuum of activation rather than discrete, fixed subsets. A refined, integrative approach to understanding eosinophil heterogeneity is critical for improving patient stratification, predicting therapeutic responsiveness, and optimizing precision medicine strategies in chronic airway diseases.

## 1. Introduction

Eosinophils are terminally differentiated granulocytes that are classically associated with the innate immune response [1,2,3]. First described by Paul Erlich in 1879, these cells were named for their characteristic uptake of the bright red dye eosin [4]. Eosinophils develop from bone marrow-derived CD34+ hematopoietic precursor cells and are regulated by key transcription factors that include CCAAT/enhancer binding protein (C/EBP), GATA-1, and PU.1 [5]. The main cytokines that influence eosinophil maturation and development include interleukin (IL)-3, IL-5, and granulocyte-macrophage colony stimulating factor (GM-CSF), with IL-5 being regarded as the most important cytokine for eosinophilopoiesis [2,3,5]. Once activated, eosinophils release a broad repertoire of cytokines, chemokines, granule proteins, and lipid mediators that drive airway inflammation and tissue injury [1,5,6]. A defining feature of eosinophils is their ability to store preformed cytotoxic mediators within intracellular granules, which enables a fast and robust inflammatory response upon activation [7,8,9]. The most notable granule proteins include major basic protein (MBP), eosinophil peroxidase (EPX), eosinophil cationic protein (ECP), eosinophil-derived neurotoxin (EDN), and galectin-10/Charcot-Leyden Crystal (CLC) protein [9]. Further, eosinophils on specific stimuli undergo eosinophil extracellular trap death (EETosis), a mode of degranulation where histone-coated DNA webs are expelled from the eosinophil, studded with these cationic granules [10,11,12,13]. While this serves as a defense mechanism for micro/macro-organisms, the nonspecific deposition of cytotoxic granule proteins leads to collateral damage to host tissues and exacerbate inflammatory pathology in airway diseases [14].

### 1.1. Eosinophils in Airway Diseases

Asthma and chronic obstructive pulmonary disease (COPD) are two of the most prevalent chronic airway diseases, with multiple inflammatory phenotypes and endotypes observed across patient populations [15,16,17]. A significant subset of patients with asthma and COPD (50–60%) exhibit type 2 (T2)-high inflammation and elevated airway eosinophils (>3%), associated with reduced lung function, increased disease burden, and higher exacerbation risk [18,19,20,21,22]. Consequently, much of the recent mechanistic and clinical research has focused on evaluating eosinophil-targeting biologics. In these populations, normalization of sputum eosinophils, often used as a surrogate of airway tissue eosinophilia, is associated with improvements in exacerbations in asthma [23,24,25,26] and COPD [27].

Although airway inflammation is a central contributor to disease, the dominant inflammatory pathway may vary between individuals, time-points, and diseases [17,28,29]. Following an allergen challenge, eosinophil recruitment has been associated with the development of the late asthmatic response (LAR), occurring in 30–50% of asthma patients [30], implicating eosinophils in allergen-induced airway inflammation [31]. Blockade of upstream epithelial cytokine thymic stromal lymphopoietin (TSLP) with Tezepelumab attenuated the LAR in patients with mild allergic disease, which was associated with a reduction in sputum eosinophil counts [32]. However, more recent investigation with benralizumab (anti-IL-5Rα) that directly targets eosinophils demonstrates that the LAR persists despite the near-complete depletion of sputum eosinophils, suggesting that eosinophils themselves may not be the primary causal drivers of this response as previously thought [30,33]. Hence, even if the patient has a history of eosinophilia or has current evidence of eosinophils, these cells may not be the underlying driver of airway pathologies in asthma and COPD. Clinical trial data indicate that despite achieving comparable reductions in blood eosinophil counts, patients with COPD were observed to experience a smaller magnitude of reductions in annual exacerbations [34,35] compared to asthma patients [25,26]. When stratified by degree of eosinophilia, the MATINEE (NTC04133909, 100 mg mepolizumab subcutaneous Q 4 weeks) and BOREAS (NTC03930732, 300 mg dupilumab subcutaneous Q 2 weeks) trials demonstrated that patients with COPD that present with blood eosinophilia (>300 cells/μL) experience significant reductions in exacerbations following anti-IL-5 [36] or anti-IL-4/13Rα therapy [37].

In addition to therapies that directly target eosinophils, biologics that interfere with alternate components of the type-2 inflammatory cascade (beyond canonical IL-5 signalling) have provided further insight into eosinophil biology. Blockade of IL-4 and IL-13 signaling with dupilumab in an unselected moderate-to-severe asthma population has been shown to reduce exacerbations in severe asthma by 47% (NTC02414854, 200 or 300 mg dupilumab subcutaneous Q 2 weeks) [38] and oral corticosteroid use by 70% (NTC02528214, 300 mg dupilumab subcutaneous Q 2 weeks) [39]. Mechanistically, IL-4 and IL-13 stimulate epithelial and stromal cells to produce eotaxins (CCL11, CCL24, CCL26), which promote eosinophil chemotaxis via the CCR3 receptor expressed on circulating eosinophils [40,41]. IL-4 and IL-13 also promote upregulation of vascular cell adhesion molecule-1 (VCAM-1), facilitating eosinophil adhesion to the endothelium and subsequent transmigration into the tissues [41].

Notably, transient increases in peripheral eosinophil counts have been reported in 4–14% of dupilumab-treated patients, which may reflect altered eosinophil trafficking mechanisms into tissues [38,39], while a moderate population has shown reduction in sputum eosinophils [42]. In this context, eosinophils that accumulate in the circulation during dupilumab treatment may represent cells that are no longer efficiently recruited into the airways and thus may not directly contribute to airway inflammation. Conversely, eosinophils that persist within the airways despite IL-4/IL-13 blockade may represent a distinct and clinically relevant population maintained through IL-4/IL-13-independent pathways. Rare eosinophilic complications have also been described following initiation of dupilumab therapy, including cases suggestive of previously masked antineutrophil cytoplasmic antibody (ANCA)-negative eosinophilic granulomatosis with polyangiitis (EGPA) [43]. Collectively, these observations highlight that eosinophil biology in disease may be shaped not only by the cell numbers but also by migration, tissue localization, and disease state. Determining the phenotype and functional properties of eosinophils that persist in circulation versus those that remain within airway tissues during biologic therapy represents an important unresolved question, with implications for understanding treatment responses and identifying biologic-unresponsive eosinophil populations.

Together, these findings emphasize the need for careful patient stratification, highlight that eosinophils may contribute to disease activity in patients differentially across diagnosis, and demonstrates that evidence of eosinophils does not ascertain them as primary drivers of symptoms. Such findings support the concept that functional heterogeneity among eosinophils may differentially influence disease pathogenesis, symptom control, and treatment responsiveness.

### 1.2. Eosinophil Heterogeneity

Eosinophil heterogeneity refers to the existence of distinct eosinophil subpopulations within the immune system that exhibit diverse phenotypes and perform specialized functions that may be compartmentalized (tissue vs. blood). Heterogeneity may be driven by host characteristics such as age and biological sex. Circulating eosinophil counts and functional responses vary across the lifespan, and eosinophils are suggested to be less prone to activation with increased age [44]. Age-related differences may also extend to the airway microenvironment. Studies in preschool wheeze and school-age asthma demonstrate early alterations in the extracellular matrix composition, including increased collagen deposition and lumican expression, which contribute to airway remodeling and may influence inflammatory responses within the tissue [45]. Emerging proteomic studies further suggest that sex may modify inflammatory responses to aeroallergen exposure, indicating potential sex-specific regulation of eosinophil effector function [46]. In addition to host factors, environmental factors such as noxious chemicals and particulate matter are associated with elevated blood eosinophils and may contribute to the development of chronic airway diseases [47].

Early evidence for eosinophil heterogeneity emerged from studies of patients with hypereosinophilic syndromes (blood eosinophils > 400 cells/μL), where distinct hypodense eosinophil populations were identified [48,49,50,51,52]. These cells exhibited enhanced metabolic activity, altered granule morphology, and increased effector functions compared to normodense eosinophils, providing the initial evidence that eosinophil activation may have a distinct cellular morphology. Subsequent work extended these observations to tissue-resident eosinophils, which were shown to persist in healthy states in the small intestine [53,54], colon and cecum [55], skin [56], and lungs [57,58,59]. Indeed, tissue-resident eosinophils have been reviewed extensively [60,61].

Building on these studies, surface marker-based approaches is now widely used to define eosinophil subsets in both experimental models and human patients. Of note, a study of murine lung tissue identified resident and inflammatory eosinophils distinguished by differential expression of the L-selectin protein, CD62L, with parallel validation in human eosinophilic asthma patients [62], suggesting that discrete eosinophil subsets may underlie divergent functional roles in airway disease. Although the present review will focus on airway and allergic diseases, eosinophil heterogeneity is also well-characterized in gastrointestinal disorders where eosinophils within the small intestine and colon play established roles in host defense and tissue homeostasis [53,54,55,60]. These studies similarly suggest that eosinophils adopt context-dependent activation states, supporting the concept that eosinophil heterogeneity represents a broader feature of eosinophil biology, rather than a phenomenon unique to airway inflammation.

In this review, we synthesize current evidence on eosinophil heterogeneity in airway diseases with a focus on the strengths and limitations of surface marker-based classification, and the insights gained from high-throughput molecular profiling. We argue that eosinophil diversity is best understood as a spectrum of context-dependent cellular states than discrete, fixed subsets, and that integrative approaches combining transcriptomics, proteomics, and functional assays are essential to fully define eosinophil biology in health and disease. By reframing eosinophil heterogeneity in this manner, this review aims to provide a conceptual framework of eosinophil activity and link eosinophil molecular programs to tissue-specific function and pathology.

## 2. Surface Marker-Defined Eosinophil Heterogeneity

As interest in eosinophil heterogeneity increases, a growing body of literature has examined eosinophil subpopulations in airway diseases using flow cytometry-based surface marker profiling. These studies have primarily been of adult asthma patients, assessing allergic/extrinsic and severe eosinophilic/intrinsic asthma [63,64,65,66,67,68,69,70,71]. These studies are summarized in Table 1. To date, only two studies have assessed eosinophil heterogeneity with surface markers in COPD patients [72,73] and only one in pediatric asthma patients [74].

### 2.1. Surface Markers Reflect Eosinophil Activation and Maturation States

Eosinophils express a distinct set of surface markers that differentiates them from other granulocytes [2,3]. Classical identifiers such as Sialic acid-binding immunoglobulin-like lectin (Siglec)-8 and CC-chemokine receptor (CCR)-3 remain foundational for eosinophil detection [75,76]. More recent efforts to characterize eosinophil heterogeneity have expanded beyond these canonical markers to capture functional diversity, such as CD63 for degranulation-associated activation, and CD66b (CEACAM-8), a neutrophil-associated activation marker that has been observed on activated eosinophil subsets. Additional markers, such as CD101, have further refined eosinophil classification: CD101^lo^ eosinophils express regulatory phenotypes, whereas CD101^hi^ eosinophils align with proinflammatory activity [62]. Studies of acute lung injury and acute respiratory distress syndrome have identified CD101^lo^ eosinophils capable of suppressing neutrophilic inflammation [77], with similar populations proposed in eosinophilic esophagitis [78].

**Table 1:** Surface marker subtyping of eosinophils by study (see Figure 1 for diagram of eosinophil surface marker expression and biological functions).

Study	Disease	Compartment	Markers Used to Subtype Eosinophils	Defined Eosinophil Populations
[63]	Mild AA patients Severe EA patients	Peripheral blood	CD62L, CD101	rEos: CD62L^hi^CD101^lo^ iEos: CD62L^lo^CD101^hi^
[64]	Mild AA patients	Peripheral blood	CD62L	rEos: CD62L^hi^ iEos: CD62L^lo^
[65]	Mild AA patients	Peripheral blood	CD62L	rEos: CD62L^hi^ iEos: CD62L^lo^
[66]	Mild AA patients	Peripheral blood	CD62L	rEos: CD62L^hi^ iEos: CD62L^lo^
[67]	Mild-moderate AA patients	Induced sputum	CD15, CD66b	E1: CD15+ CD66b+ E2: CD15− CD66b−
[68]	Severe EA patients	Peripheral blood Nasal polyp tissue	CD62L	rEos: CD62L^hi^ iEos: CD62L^lo^
[69]	Severe EA patients	Peripheral blood	CD62L	rEos: CD62L^hi^ iEos: CD62L^lo^
[70]	Severe EA patients	Peripheral blood	CD62L	rEos: CD62L^hi^ iEos: CD62L^lo^
[71]	Severe EA patients	Peripheral blood	CD62L	hEos: CD62L^hi^ iEos: CD62L^lo^
[72]	Mild-moderate asthma and COPD patients	Peripheral blood Induced sputum	CD11b, CD14, CD62L, CD66b, CD125, CD193	1: CD125−CD193+ 2: CD125+CD193+ 3: CD66b+CD193+
[73]	Mild-severe asthma and COPD patients	Peripheral blood	Siglec-8, CD62L, CD123	rEos: Siglec-8^+^CD62L^hi^CD123^lo^ iEos: Siglec-8^+^CD62L^lo^CD123^hi^
[74]	Severe EA patients *	Induced sputum	CD62L	C1-C4: CD62L^lo^ C6 and C7: CD62L^int^ C5 and C8: CD62L^hi^
[79]	CRSwNP Patients	Nasal polyp tissue	CD62L	rEos: CD62L^hi^ iEos: CD62L^lo^

Abbreviations: AA, allergic asthma; C, cluster; COPD, chronic obstructive pulmonary disease; CRSwNP, chronic rhinosinusitis with nasal polyps; EA, eosinophilic asthma; hEos, homeostatic eosinophils; iEos, inflammatory eosinophils; rEos, resident eosinophils. * Pediatric patients.

**Figure 1:**
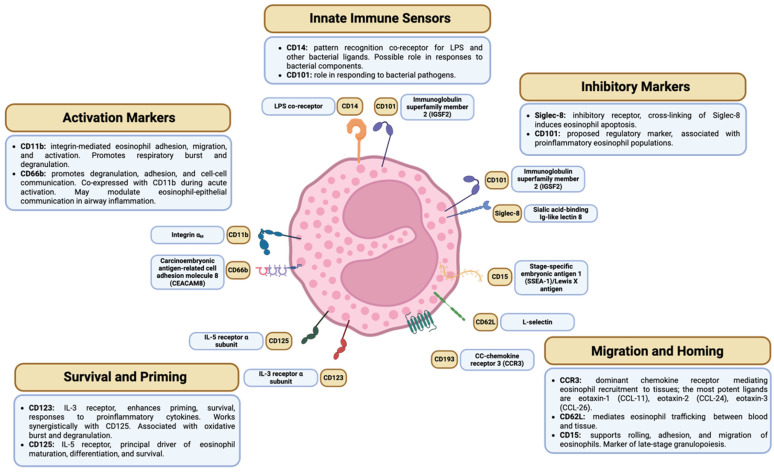
Select surface receptors of human eosinophils for subtyping and associated biological function. Schematic representation of select key cytokine receptors, chemokine receptors, adhesion molecules, innate immune sensors, and inhibitory receptors used to stratify eosinophils into distinct subpopulations. Differential expression of CD123 and CD125 reflects survival and priming capacity; CCR3, CD11b, CD62L, and CD15 report migratory and tissue-homing programs; CD66b indicates eosinophil activation and degranulation; CD14 and CD101 denote innate immune sensing; Siglec-8 and CD101 promote regulatory and inhibition of eosinophil populations. Together, these markers have been used to provide a framework for classifying discrete eosinophil subtypes across blood and tissue compartments in allergic and airway diseases. Adapted from [2,3]. Abbreviations: CD, cluster of differentiation; IL, interleukin; LPS, lipopolysaccharide. Created in https://biorender.com.

Figure 1 summarizes the principal markers used in assessing eosinophil heterogeneity; within this review, particular emphasis is placed on studies that evaluate CD62L/L-selectin in eosinophil subtyping.

CD62L, otherwise known as L-selectin, is widely expressed on leukocytes including eosinophils, neutrophils, lymphocytes, and monocytes, and is responsible for facilitating leukocyte rolling and adhesion to the surface of the endothelium to facilitate tissue extravasation via sialyl Lewis X glycans [80]. Of all surface proteins, CD62L is most frequently used to stratify eosinophil subpopulations in airway diseases, with CD62L^lo^ eosinophils generally considered inflammatory, and CD62L^hi^ proposed to be regulatory or homeostatic [62].

CD62L^lo^ eosinophils exhibit features consistent with activation, including elevated expression of CD69 [68] and increased spontaneous production of reactive oxygen species (ROS) in allergic asthma [66]. Patients with more severe asthma also demonstrate increased ROS production across eosinophil subtypes compared to patients with mild disease and healthy controls, suggesting overall eosinophil dysregulation, rather than subtype-specific effects alone that modulate disease severity [66].

In contrast, CD62L^hi^ eosinophils express increased costimulatory molecules such as CD28 and CD86 compared to CD62L^lo^ populations [68], which supports their potential regulatory and homeostatic role respectively. For other key surface receptors, such as the IL-5 receptor α subunit (CD125) that binds soluble IL-5, evidence supporting its role in eosinophil heterogeneity is largely inconsistent. In one study of mild-to-severe asthma and COPD patients, CD125 was enriched in CD62L^lo^ eosinophil populations [72]. A subsequent study of severe asthma patients with eosinophilia revealed higher expression of CD125 in CD62L^hi^ populations [68], while another study showed no differences across CD62L^lo^ and CD62L^hi^ populations [71], highlighting unresolved heterogeneity in maturation and cytokine sensitivity. Together, these conflicting data across studies suggest that surface markers reflect eosinophil activation and maturation states, but individual markers alone may be unable to define stable or functionally distinct populations.

### 2.2. Compartment-Specific Eosinophil Phenotypes

Eosinophils differ markedly across biological compartments, reflecting their tissue-specific localization and the influence of local microenvironmental signals. For instance, in asthma, CD11b expression is significantly elevated on sputum eosinophils compared to blood eosinophils, a difference that was not observed in COPD patients [72]. Similarly, eosinophils that co-express CD66b and CD193 are enriched in the sputum of COPD patients compared to asthma patients, despite CD66b expression alone not differing by compartment or disease [72].

There is strong evidence to suggest compartmental differences among eosinophils in upper airway diseases. In patients with severe asthma and comorbid chronic rhinosinusitis with nasal polyps (CRSwNP), nasal polyp tissue is enriched in CD62L^lo^ eosinophils compared to the peripheral blood [62,68,79]. Nasal polyp eosinophils express less CD193, and more CD69 than circulating eosinophils [69]. Notably, CD69 expression on nasal polyp populations negatively correlates with forced expiratory volume in 1 s (FEV1) in CRSwNP patients [81]. The proportion of CD62L^lo^ eosinophils in nasal polyps correlates positively with disease burden measured by Sino-Nasal Outcomes Test (SNOT)-22 scores (r = 0.36, *p* < 0.01) [69].

Notably, compartment-dependent differences in CD62L expression are not unique to eosinophils themselves. Similar patterns have been observed among other immune cells, such as in neutrophils and innate lymphoid cells (ILCs). Resting neutrophils express CD62L, and stimulation results in the shedding of CD62L from the neutrophil cell membrane [82]. CD62L^lo^ neutrophils are enriched in the tissue compared to circulation and are proposed to possess inflammatory functions [82,83]. This was further explored in pediatric patients with recurrent severe wheeze, where airway neutrophil phenotypes were strongly compartmentalized. CD62L^lo^ neutrophils predominated in bronchoalveolar lavage fluid of children in the infection-predominant cluster, while CD62L^hi^ neutrophils were enriched in children without an airway infection; these specific neutrophil clusters were not identified in systemic neutrophils [84]. Similarly, other studies report CD62L expression on inactive ILC precursors and demonstrate loss of CD62L in tissue ILCs compared to those in the peripheral blood [85,86]. Taken together, these findings suggest that CD62L modulation likely occurs due to tissue trafficking and activation, rather than eosinophil-specific lineage commitment.

### 2.3. Associations with Disease Severity and Clinical Outcomes

To assess their potential clinical relevance, multiple groups have evaluated if eosinophil subpopulations defined by surface markers associate with measures of disease activity, control, and exacerbations in asthma and COPD patients. In severe asthma, circulating CD62L^lo^ eosinophils negatively correlate with Asthma Control Test (ACT) scores (r = −0.36, *p* < 0.01) and positively correlates with scores from the Asthma Control Questionnaire (ACQ) (r = 0.35–0.40, *p* < 0.05) [69,71]. Lower ACT scores, and higher ACQ scores indicate poor asthma control, suggesting that expansion of CD62L^low^ eosinophils is associated with poor disease control. In contrast, CD62L^hi^ eosinophils negatively correlate with ACQ scores (r = −0.38, *p* < 0.05) [71], consistent with the proposed non-pathogenic role of CD62L^hi^ eosinophils [62].

Additional focus has been given to severe asthma patients with concomitant CRSwNP. In this subpopulation, enrichment of CD62L^lo^ eosinophils in nasal polyp tissue is associated with increased symptom burden [68,69,79], reinforcing the link between clinical outcomes and tissue-localized eosinophils. No conclusive data on CD62L^hi^ eosinophils and nasal polyp burden is available, and it remains to be seen whether enrichment of CD62L^hi^ eosinophils in nasal polyp tissue is associated with better clinical outcomes.

One area that deserves further investigation is the role of eosinophil subpopulations and control of COPD. To date, no associations have been found between eosinophil subtypes and COPD control measured by the COPD Assessment Test (CAT), nor across severity grades outlined by the Global Initiative for Obstructive Lung Diseases (GOLD) [72,73]. This presents as a major gap in current knowledge and warrants further exploration of the association between eosinophil subpopulations and COPD outcomes.

The supporting evidence for linking eosinophil subtypes to exacerbation risk is largely inconsistent. Several studies of asthma and COPD report no association between exacerbation frequency and eosinophil subtypes [67,73]. In contrast to these findings, Vultaggio and colleagues observed a weak but positive correlation between circulating CD62L^lo^ eosinophils and the annual exacerbation rate in severe asthma (r = 0.36, *p* < 0.01) [69]. Pediatric studies further complicate interpretation, as Wilson and colleagues identified CD62L^hi^ eosinophils as being enriched in pediatric asthma patients who experienced at least one exacerbation in the year prior to the study onset [74]. Compartmental differences may be one factor contributing to the observed differences, as Vultaggio and colleagues used blood eosinophils, whereas Wilson and colleagues collected eosinophils from sputum. Additionally, age-related differences may explain the discrepancies, as eosinophils in younger individuals exhibit greater capacity to degranulate in response to immunogenic stimuli [44,87]. Notably, across the studies that evaluated demographic variables, no consistent associations were identified between biological sex and eosinophil subpopulation distribution or their relationship with exacerbation risk. Taken together, these findings indicate that eosinophil subpopulations and clinical disease activity are context-dependent, may vary by age, disease, and compartment, and remain insufficiently consistent to support their use as biomarkers of disease activity.

### 2.4. Modulation by Corticosteroids and Biologic Therapies

Oral corticosteroids exert profound effects on eosinophil biology and can rapidly reduce circulating eosinophils through suppression of eosinophil production in the bone marrow and induction of eosinophil apoptosis. They also impair eosinophil activation and trafficking to inflamed tissues by suppressing type-2 inflammatory signaling pathways. Importantly, corticosteroids act broadly across eosinophil subpopulations, suppressing cells maintained by diverse signaling pathways rather than selectively targeting those dependent on a single axis. This pan-suppressive effect may reduce both inflammatory and tissue-resident eosinophil subsets. Any differential impact of corticosteroids on eosinophil subpopulations remains unclear. Despite the widespread use of inhaled corticosteroids (ICS) as standard of care and common inclusion in clinical investigations, neither ICS, nor oral corticosteroid (OCS) use has been consistently associated with specific CD62L-defined subtypes in asthma or COPD [63,64,65,66,69,70,71,73].

In contrast, biologic therapies that target IL-5 signaling exert marked effects on CD62L-defined eosinophil subpopulations. Treatment with mepolizumab reduced circulating CD62L^lo^ eosinophils while preserving the CD62L^hi^ population [69,71,74], suggesting that CD62L^lo^ eosinophils are dependent on IL-5 for survival, whereas CD62L^hi^ populations maintain survival via IL-5-independent mechanisms. Targeting the IL-5 receptor with benralizumab completely abrogates both populations and eliminates CD62L^hi^ eosinophils that persist with mepolizumab treatment [71]. These findings highlight that pathway-specific biologic therapies may differentially affect eosinophil subpopulation and provide mechanistic insight into eosinophil heterogeneity.

Post-biologic therapy, reductions in CD62L^lo^ eosinophils correlate with improved asthma control, which is reflected in increases in ACT scores and reduced ACQ scores [69,70,71]. In patients with severe asthma and comorbid CRSwNP, mepolizumab reduces both circulating and nasal polyp CD62L^lo^ eosinophils, which was accompanied by improvements in SNOT-22 scores [79]. At the cellular level, these clinical benefits are paralleled by reduced expression of cytotoxic granule proteins, including major basic protein (MBP), eosinophil peroxidase (EPX), eosinophil cationic protein (ECP), eosinophil-derived neurotoxin (EDN), and galectin-10/Charcot-Leyden crystal protein (CLC) across both CD62L^hi^ and CD62L^lo^ populations [70], indicating that biologic therapies may attenuate disease by suppressing effector function in eosinophil subpopulations. Biologics targeting additional type-2 cytokines may further influence eosinophil trafficking and localization. For example, blockade of IL-4 and IL-13 with dupilumab has been associated with transient increases in circulating eosinophils, likely reflecting reduced migration of eosinophils from circulation into the tissues [38,39]. Eosinophil heterogeneity appears to be modulated by biologic therapies, and future studies are needed to clarify the functional consequences of these treatments on eosinophil subpopulations.

## 3. Limitations of CD62L-Based Subtyping of Eosinophils

To date, eosinophil heterogeneity has been largely defined by surface marker expression, most commonly L-selectin/CD62L, initially identified in murine models of allergic airway inflammation [62]. While CD62L-based classification has provided useful insight into inflammatory and tissue resident eosinophils, this approach is insufficient to fully capture eosinophil heterogeneity in human airway diseases.

A key limitation of CD62L-based classification is its reliance on murine models, which differ substantially from human eosinophil biology. Murine eosinophils do not undergo degranulation in response to allergen challenges [88], and granule proteins such as MBP and EPX do not contribute to allergen-induced airway inflammation in this model [89,90]. In addition, murine eosinophils lack expression of the CLC protein, a key eosinophil effector protein in human eosinophil biology [91]. These considerations limit the translatability of CD62L-defined subsets across species.

Surface marker expression alone does not capture the full biological complexity and functionality of this cell type. Distinct eosinophil populations may share overlapping surface markers yet differ substantially in their effector functions, activation states, or gene expression profiles. As a result, reliance on surface markers such as CD62L risks grouping functionally divergent eosinophils into the same subtype, which obscures biologically meaningful heterogeneity. Additionally, CD62L modulation is not unique to eosinophils and occurs across multiple leukocyte populations in response to activation and tissue trafficking, limiting its specificity of eosinophil-intrinsic heterogeneity.

Finally, CD62L-based eosinophil populations demonstrate limited and inconsistent clinical relevance. Demographic variables such as age, biological sex, body mass index, smoking history, and age of disease onset have not been shown to associate with CD62L-defined eosinophil subsets. Moreover, these populations do not exhibit stable or consistent co-expression of other surface markers, limiting their utility as disease biomarkers. Although associations between CD62L and poor disease control have been reported, these correlations remain modest at best. There is also no evidence available to suggest that corticosteroid treatment differentially affects CD62L-defined eosinophil subpopulations, and differential effects of T2 biologics have been reported in other cell types such as ILCs [92]. Data regarding enrichment of CD62L-defined populations during exacerbations are likewise inconclusive. Collectively, these limitations highlight the need for alternative approaches to define eosinophil heterogeneity to more accurately reflect disease mechanisms and predict therapeutic responses.

## 4. Transcriptomic/Proteomic Approaches to Eosinophil Heterogeneity

Transcriptomic and proteomic approaches present high-resolution, unbiased characterization of eosinophil states that extend beyond conventional surface marker-based phenotyping. These methods consistently reveal functional heterogeneity across diseases and compartments that is not apparent with immunophenotyping strategies, underscoring their importance for understanding the complexity of eosinophil biology in airway and allergic diseases [6] (Table 2). However, most transcriptomic studies have been performed on peripheral blood eosinophils, which may not fully reflect the transcriptional states of airway or tissue-resident eosinophils. Consequently, clustering analyses based on circulating eosinophils may underestimate the full spectrum of eosinophil heterogeneity within diseased airways. Local microenvironmental cues, including cytokines and cell–cell interactions, can induce transcriptional and phenotypic plasticity in immune cells; this was demonstrated by Ju and colleagues, where IL-1β and IL-18 in sputum drove type-2 ILCs towards an intermediate type-2/3 ILC phenotype in severe asthma [93]. Such microenvironment-driven plasticity is unlikely to be captured in the blood, highlighting the importance of profiling tissue-resident populations. Similarly, each time an eosinophil crosses a tissue barrier, it becomes increasingly activated and alters surface marker expression; thus, eosinophils recovered from the airway lumen, such as in sputum, represent activated forms that are more relevant to airway disease pathology than their circulating counterparts.

### 4.1. Transcriptional and Proteomic Heterogeneity

Single-cell transcriptomic studies across allergic diseases demonstrate that circulating eosinophils are by and large, transcriptionally homogenous and functionally quiescent, even in the setting of active disease; in contrast, tissue eosinophils acquire distinct activation patterns shaped by cues from the local microenvironment. However, single-cell RNA sequencing (scRNA-seq) in eosinophils remains technically challenging due to low RNA and high ribonuclease content, susceptibility to activation during isolation, and fragility during standard single-cell processing workflows. As a result, the number of high-resolution human transcriptomic datasets available for eosinophils remains limited compared to other immune cell types.

In eosinophilic esophagitis (EoE), circulating eosinophils from patients with active disease and healthy controls share highly overlapping transcriptional profiles, whereas tissue eosinophils (only found in EoE patients) segregate into distinct populations enriched in activation, proinflammatory processes, and survival pathways, indicating tissue-specific instruction (Table 2) [94]. Similarly, in chronic rhinosinusitis patients, nasal polyp eosinophils display extensive transcriptional divergence from the circulating population, including upregulation of activation, anti-apoptotic, and NF-κB-related genes, and downregulation of genes involved in leukocyte migration and adhesion (Table 2) [95]. These tissue-derived transcriptional states can be driven by stimulation of blood eosinophils in vitro with IL-1β and IL-33, reinforcing the concept that heterogenous eosinophil populations reflect stimulus-dependent plasticity rather than discrete, fixed subsets [95].

These findings are further supported by transcriptional analyses. Bulk RNA profiling demonstrated that circulating eosinophils in moderate-to-severe asthma are not intrinsically proinflammatory, and instead are enriched in processes involved in homeostasis, tissue repair, and cell migration (Table 2) [96]. Subsequent comparison of these transcriptional signatures with circulating eosinophils from hypereosinophilic disorders demonstrates a high degree of conservation, suggesting a shared circulating eosinophil profile despite differences in disease [96]. More recent single-cell studies identified multiple clusters of circulating eosinophils in the peripheral blood [97,98]. Rather strikingly, most clusters were shared between patients with asthma and healthy controls [97,98]. Disease-associated differences were subtle and context-dependent, with some studies reporting specific clusters enriched in asthma and associated with worse lung function and elevated T2 inflammation [97], and others demonstrating severity-dependent transcriptional differences, which is characterized by elevated interferon-stimulated gene signatures in severe asthma compared to milder forms of disease (Table 2) [98]. These findings collectively indicate that circulating eosinophils exhibit graded, rather than discrete, variation in transcriptional signatures.

Emerging single-cell transcriptomic analyses of airway-derived samples further highlights the complexity of eosinophil heterogeneity in human disease. scRNA-seq profiling of sputum and lung biopsy samples from patients with severe asthma identified eosinophils based on a conserved transcript signature of *EPX*, *CCR3*, *CD101*, *CLC*, *SELL*, *ANXA1*, *SIGLEC10*, *ADGRE1*, *ITGAX*, *ITGB2*, and *ALOX15* [99]. However, identifiable eosinophil subtypes were not observed in sputum nor biopsy specimens. These findings suggest that eosinophil heterogeneity in human airway disease may be context-dependent and influenced by local microenvironmental signals rather than representing discrete transcriptionally defined subsets. Several of these genes, including *EPX*, *CLC*, and *ALOX15* are highly enriched in eosinophils and are commonly used to identify eosinophils in bulk and single-cell transcriptomic datasets. However, similar to surface marker expression, few genes are exclusively expressed by eosinophils, and most are shared at lower levels with other granulocytes such as neutrophils and basophils. Consequently, eosinophil identification in transcriptomic studies typically relies on combinations of enriched gene signatures rather than a single eosinophil-specific marker.

Comparative transcriptomic analyses between asthma and COPD patients further emphasize the limited disease specificity of circulating eosinophils. Although modest differences in gene expression patterns have been identified, including the enrichment of macrophage inflammatory protein isoforms *CCL3L1* and *CCL4L2* in patients with moderate COPD, the transcriptional overlap in circulating eosinophils between the two diseases remains substantial [100]. This suggests that disease-specific eosinophil functions are driven by signals within the diseased tissue rather than be intrinsically part of circulating populations (Table 2) [100]. This is reinforced by investigation of COPD patients stratified by blood eosinophil counts (>150 cells/μL: eosinophilic; <150 cells/μL: non-eosinophilic) [101]. This study demonstrated that distinct transcriptional and proteomic signatures are associated with eosinophilic and non-eosinophilic states, such as elevated antiviral pathways and antibacterial mechanisms, respectively (Table 2) [101].

Evidence of eosinophil plasticity is further supported by single-cell analyses of nasal lavage fluid from pediatric patients, which reveal eosinophil subpopulations enriched for neutrophil-associated transcriptional programs in type 2-low disease [102]. These findings parallel observations in non-eosinophilic COPD [101] and are complimented by the identification of subpopulations of sputum neutrophils that express eosinophil-associated genes in asthma with type 2-high inflammation (Table 2) [102,104]. In vitro stimulation of neutrophil precursors with IL-5 induces expression of eosinophil-associated receptors, thereby supporting the concept of granulocyte plasticity in airway inflammation [104].

Profiling of eosinophils with transcriptomic and proteomic approaches has also provided insight into the impact of biologic therapies on eosinophil states. In severe asthma, mepolizumab treatment downregulates IL-5-signaling pathways and induces expression of interferon-associated and immune regulatory genes, without any alteration to CD62L expression (Table 2) [103]. These findings contrast with surface marker-based studies and suggest that biologic therapies may reshape eosinophil functional programs rather than simply depleting pathogenic subsets. Murine studies demonstrating stimulus-specific modulation of eosinophil transcriptional profiles further supports this continuum model of eosinophil activation [105,106]. Notably, transcriptomic studies in murine models suggest that tissue residency itself may be a key determinant of eosinophil transcriptional programs, functional specialization, and heterogeneity [107]. However, comparable high-resolution transcriptomic analyses of tissue eosinophils in human airway disease remain limited. Eosinophils recovered from the airway lumen, such as those in sputum or bronchoalveolar lavage (BAL), have crossed an additional epithelial barrier and are thus in a further activation state. These cells may reveal transcriptional and functional programs that are directly relevant to airway disease pathology.

### 4.2. Bridging Gaps in Transcriptomic and Proteomic-Defined Eosinophil Heterogeneity

Collectively, transcriptomic and proteomic studies reveal a level of eosinophil heterogeneity that is not captured by surface marker expression alone. These data consistently indicate that circulating eosinophils are transcriptionally inclined for migratory processes rather than inflammation, and disease-relevant functional specialization is largely acquired within the tissue in response to local signals. Despite limitations including small sample sizes, technical challenges associated with performing molecular studies on eosinophils, and the high cost of sequencing technologies, emerging techniques that employ gentler methods of eosinophil isolation may improve the resolution and feasibility of transcriptional profiling of eosinophils [94,108]. Future work should integrate transcriptomic and proteomic approaches across biological compartments and disease states, and responses to treatments, for defining biologically meaningful eosinophil subsets to inform precision medicine.

## 5. Conclusions and Future Directions

The studies reviewed here demonstrate that eosinophils contribute to airway disease in heterogenous and context-dependent ways, reflecting underlying biological diversity among patients. Although stratification by flow cytometry into CD62L^hi^ and CD62L^lo^ populations have underscored most efforts to define eosinophil heterogeneity and have been linked to clinical outcomes such as disease control and exacerbation risk, molecular profiling studies reveal that heterogenous eosinophil populations are far more complex. Transcriptomic and proteomic analyses demonstrate that eosinophils exhibit distinct molecular programs that cannot be captured by binary surface marker classification, and eosinophil heterogeneity may be better conceptualized as a spectrum of activation and functional states rather than a small number of discrete subsets. Despite these advances, our understanding of eosinophil heterogeneity in airway diseases remains in its early stages, and many aspects of eosinophil biology, including how heterogeneity arises and whether it directly influences disease pathogenesis remain incompletely understood.

Current models of eosinophil heterogeneity are hindered by major gaps in study design and sampling approaches. Most studies rely on cross-sectional sampling that limits insight into how eosinophil populations evolve during disease progression, exacerbations, and therapeutic interventions. This is particularly problematic given that corticosteroids and biologics are known to profoundly impact eosinophil biology, and future groups will want to investigate how eosinophil subpopulations are modulated by these therapies. In addition, most investigations are restricted to eosinophils from the peripheral blood, obscuring how eosinophils are reprogrammed as they traffic into inflamed tissues, where local cues from the microenvironment modulate infiltrating eosinophils. Finally, eosinophil heterogeneity in COPD remains markedly understudied, despite growing evidence that eosinophils contribute to COPD pathobiology differently than in asthma and may influence responsiveness to therapeutics such as biologics.

A major unresolved challenge is linking eosinophil heterogeneity to functional outcomes. Since eosinophils that mediate disease-relevant activities are most often located within tissues, direct functional assessment of defined eosinophil subsets remains technically difficult. As a result, many studies infer functional differences indirectly by integrating surface marker expression, transcriptional signatures, and correlations with disease states rather than through direct functional assays.

Emerging evidence supports a model in which eosinophil heterogeneity reflects a continuum of activation and functional phenotypes shaped by tissue-specific signals rather than fixed lineages. Cytokine-driven eosinophil plasticity has been demonstrated in vitro [104,105,109,110,111,112], yet how these transcriptional and functional programs are orchestrated in vivo in airway diseases remains incompletely understood. As eosinophils traffic from the circulation into inflamed airways, they encounter a complex and dynamic microenvironment composed of epithelial, stromal, and immune cells; extracellular matrix (ECM) components; soluble mediators; and microbial communities. Each of these elements has the potential to modulate eosinophil activation, degranulation, cytokine production, survival, and contributions to tissue remodeling. Disentangling the relative influence of these signals—whether driven by ECM interactions, cytokines, or microbe-derived factors—represents a key priority for future mechanistic studies. Furthermore, emerging evidence raises the possibility that eosinophils may in some contexts act as biomarkers of underlying inflammatory processes rather than primary drivers of disease pathology. Distinguishing whether eosinophil heterogeneity reflects causal disease mechanisms or secondary responses to broader immune dysregulation will therefore represent an important objective for future research.

Moving the field forward will require a consensus-driven framework for defining eosinophil heterogeneity that extends beyond surface marker expression alone and incorporates standardized phenotypic, functional, and molecular criteria. A recent pivotal investigation of murine eosinophils established a unified framework of eosinophil biology, demonstrating eosinophil form and function are shaped by reciprocal interactions between intrinsic developmental programs and tissue-derived microenvironment cues [107]. Whether this framework is conserved in human eosinophils, and how species-specific differences in eosinophil biology shape these interactions in human airway disease remains to be determined. Nonetheless, establishing harmonized marker panels and functional readouts that can be applied consistently across biological compartments and disease contexts will be essential for enabling meaningful cross-study comparisons, accurately understanding disease biology, and supporting the development of precision medicine approaches in airway diseases.

## Figures and Tables

**Table 2:** Transcriptomic and proteomic signatures of eosinophil subpopulations across airway diseases and compartments.

Study	Disease	Compartment	Population(s) of Interest	Representative Upregulated Genes or Proteins	Implicated Biological Pathways	Clinical or Biologic Relevance
[94]	Eosinophilic esophagitis	Peripheral blood	Circulating eosinophils	*MT-ND1*, *MT-ND2*, *MT-ND3*	Cellular respiration, aerobic respiration, ATP synthesis	Circulating eosinophils are inactive despite active disease
		Esophageal tissue	Tissue eosinophils	*SUCNR1*, *HRH4*, *DNAAF1*, *OLIG2*, *CLC*, *CCR3*	Immune regulation, cell activation, leukocyte activation, regulation of apoptosis	Tissue microenvironment modulates eosinophil biology
[95]	Chronic rhinosinusitis	Peripheral blood	Circulating eosinophils	*CCL4*, *CCL4L2*, *IGKC*, *CLDN4*	Leukocyte migration, cell adhesion	Circulating eosinophils are inactive despite active disease
		Nasal polyp tissue	Tissue eosinophils	*CD44*, *CD69*, *NFKB1/2*, *IL1A*, *IL1B*, *IL1RL1*, *PTGS2*, *CXCL8*, *BCL2A1*, *BCL2L1*	Inflammatory response, NF-κB signaling pathway, cytokine signaling in immune system, negative regulation of apoptosis	Tissue microenvironment and cytokine signaling drive functional heterogeneity
[96]	Severe asthma	Peripheral blood	Circulating eosinophils	*ISG20*, *IL2RA*, *IL3RA*, *LIPA*, *S100A10*, *CCS*, *GSTT1*	Response to stimuli, homeostasis, wound healing	Circulating eosinophils are not intrinsically proinflammatory
[97]	Severe asthma	Peripheral blood	Circulating eosinophils; cluster 0	*LENG8*, *CAPN15*	ATPase activity, ion transmembrane transport, NOD pathways	Circulating eosinophils are largely transcriptionally similar; CD62L is enriched in poor disease outcomes
			Circulating eosinophils; cluster 1	*CCR3*, *ANXA1*, *SIGLEC10*, *ITGB2*, *HLA-C*	Pathogen immune defense, calcium signaling	
			Circulating eosinophils; cluster 3	*IFIT3*, *MX1*, *SP100*, *XAF1*	Interferon responses, nucleoside triphosphate response	
			Circulating eosinophils; cluster 4	*CLC*, *SELL*, *S100P*, *VIM*, *TRIR*, *CXCR4*	Granule secretion, oxidative stress, TLR signaling pathways	
[98]	Moderate-to- severe asthma	Peripheral blood	Circulating eosinophils; cluster 1	*CCL4*, *S100P*, *CLC*, *HLA-A*, *HLA-B*, *HLA-C*	Antigen processing and presentation, actin filament organization, leukocyte migration	Enrichment of cluster 3 and interferon responses in severe asthma
			Circulating eosinophils; cluster 2	*EXOC4*, *ASXL2*, *ATP11B*, *MALAT1*, *DENND1A*	Small GTPase mediated signal transduction, dendrite development	
			Circulating eosinophils; cluster 3	*MX1*, *MX2*, *IFI6*, *IFIT3*, *IFIT5*, *ISG15*, *EPSTI1*	IFN-α/γ response, defense response to virus, regulation of viral processes	
[99]	Severe asthma	Airway tissue	Sputum eosinophils	*EPX*, *CCR3*, *CD101*, *CLC*, *SELL*, *ANXA1*, *SIGLEC10*, *ADGRE1*, *ITGAX*, *ITGB2*, *ALOX15*	Immune regulation, cell activation, cytokine signaling	Sputum eosinophils can be Reliably assessed with single-cell sequencing; no distinct subtypes observed
			Lung biopsy eosinophils			
[100]	Mild-to- moderate asthma	Peripheral blood	Circulating eosinophils	*JUN*, *IFITM3*, *DUSP1*, *ZNF107*, *BCL6*	Stress responses, leukocyte migration, IL-4/13 signaling pathways, IL-6 signaling pathways	Subtle differences found in eosinophil transcriptomes between diseases suggests divergent inflammatory processes
	Mild-to- moderate COPD	Peripheral blood	Circulating eosinophils	*CCL3L1*, *CCL4L2*, *RSAD2*, *SERPINB2*, *PRSS21*	Protein metabolism, GPCR ligand binding	
[101]	Eosinophilic COPD	Peripheral blood	Circulating eosinophils	*TMEM176B*, *FCER1G*, *ALOX5*, *PTGDR2*, *SQLE*, *MVD*	Cholesterol metabolism, PI3K-Akt-mTOR signaling, NF-κB signaling pathways, regulation of viral processes	Differential gene and protein expression in circulating eosinophils between eosinophilic and non-eosinophilic COPD supports that inflammatory endotypes may be reflected in eosinophil programming
				Selectin P ligand, MX Dynamin Like GTPase 2, ORMDL sphingolipid biosynthesis regulator 3	Protein processing, cholesterol synthesis, sterol regulatory element binding protein signaling	
	Non- eosinophilic COPD	Peripheral blood	Circulating eosinophils	*ELANE*, *AZU1*, *CTSG*, *NOD2*	TNF signaling pathways, oxidative stress, PI3K-Akt signaling	
				Cathepsin G, Azurocidin 1, neutrophil elastase, myeloperoxidase, defensin alpha 1B	Antibacterial processes, glutathione metabolism, regulation of phagocytosis	
[102]	Pediatric asthma	Nasal lavage	Tissue eosinophils; cluster 0	*PLAUR*, *IKZF1*, *IL3RA*, *ANPEP*, *NR4A1*, *NR4A3*	Cell communication, cell signaling, leukocyte migration	Multiple transcriptionally distinct eosinophil and neutrophil populations observed in the airways, supporting a model in which eosinophil heterogeneity is driven by tissue-specific microenvironment cues
			Tissue eosinophils; cluster 1	*JAML*, *TMSB4X*	Cytoskeletal processes, vesicle-associated proteins	
			Tissue eosinophils; cluster 2	*CD69*, *IL5RA*, *SIGLEC10*	Granule secretion, innate immune response, membrane protein synthesis	
			Tissue eosinophils; cluster 3	*IL1R2*, *AQP9*, *CSF3R*, *SOD2*, *CXCL8*	Enrichment of neutrophil effector function?	
			Tissue neutrophils; cluster 0	*CXCR1*, *CXCR2*, *ITGB2*, *NCF4*, *RGS2*, *RNF149*	Neutrophil extracellular trap pathways, granule secretion	
			Tissue neutrophils; cluster 1	*G0S2*, *CXCL8*, *ITGAX*, *IL1B*, *NAMPT*, *CCRL2*	TNF signaling pathways, NF-κb signaling pathways, inflammatory response	
			Tissue neutrophils; cluster 3	*MX1*, *IFIT2*, *IFI44*, *ISG15*, *ISG20*, *IFITM2*, *IFITM3*	Interferon signaling, defense response to virus	
			Tissue neutrophils; cluster 4	*CD69*, *IL3RA*, *SIGLEC10*, *IKZF1*, *CD300LF*	Enrichment of eosinophil effector function?	
[103]	Severe asthma	Peripheral blood	Circulating eosinophils	*CD274*, *GBP2*, *GBP5*, *TLR6*, *ISG20*, *NFKB2*	T1 response?	Biological therapies influence eosinophil states by gene expression modulation

Abbreviations: ATP, adenosine triphosphate; COPD, chronic obstructive pulmonary disease; CLC, Charcot-Leyden crystal; GPCR, G-protein coupled receptor; GTP, guanosine triphosphate; IFN, interferon; IL, interleukin; NF-κB, nuclear factor kappa B; NOD, nucleotide oligomerization domain; PI3K, phosphoinositide 3-kinase; T1, type-1 immunity; T2, type-2 immunity; TLR, Toll-like receptor; TNF, tumor necrosis factor.

## Data Availability

No new data were created or analyzed in this study. Data sharing is not applicable to this article.
